# Mental Health and COVID-19 Pandemic Stressors Among Latina/o/x College Students with Varying Self and Parental Immigration Status

**DOI:** 10.1007/s40615-021-01218-x

**Published:** 2022-01-24

**Authors:** Laura E. Enriquez, Alberto Eduardo Morales, Victoria E. Rodriguez, Karina Chavarria, Annie Ro

**Affiliations:** 1 University of California Irvine, Irvine, CA USA; 2 California State University, Channel Islands, Camarillo, CA USA

**Keywords:** COVID-19, Mental health, Stressors, Immigration status, Undocumented, Latina/o/x, College students

## Abstract

The COVID-19 pandemic has produced significant psychological distress for college students due to the sudden proliferation of stressors. We examine whether and how self and parental immigration status contributes to Latina/o/x college students’ mental health and pandemic stressors during the initial months of the pandemic. We draw on quantitative and qualitative survey data collected in March–June 2020 with 1,600 Latina/o/x University of California undergraduate students from three self-identified groups: undocumented students, US citizens with at least one undocumented parent, and US citizens with lawfully present parents. Quantitative analyses reveal that the pandemic produced widespread negative mental health effects but the severity of these effects did not differ by self/parental immigration status. Our qualitative analyses identify common pandemic-related stressors across our three student groups (financial insecurity, COVID-19 virus concerns, academic strains, and social dynamics); however, undocumented students and US citizens with undocumented parents identify unique aspects of these stressors due to legal vulnerabilities. Self and parental undocumented status also compromises the ability to manage common pandemic stressors because of immigration status-related exclusion from necessary resources. Ultimately, we argue that the high-stress nature of the pandemic elevated mental distress across all student groups, but the structural exclusion of undocumented immigrants contributes to unique experiences of stress among Latina/o/x undocumented students and US citizen students with undocumented parents.

## Introduction

The COVID-19 pandemic has produced significant levels of psychological distress. A meta-analysis established a high prevalence of depression (33.7%), anxiety (31.9%), and stress (29.6%) among the general population during its initial months [1]. College students also display elevated rates of anxiety and depression compared to pre-pandemic levels [2, 3]. A study of 2,031 college students in Texas found that 48.14% expressed moderate-to-severe depressive symptoms and 38.48% expressed moderate-to-severe anxiety symptoms [4]. In a study of first-year college students in North Carolina, the prevalence of moderate-severe anxiety increased from 18.1% before the pandemic to 25.3% four months after the pandemic began, while moderate-severe depression increased from 21.5 to 31.7% [5]. While this line of research establishes severe mental health strain among college students, relatively less work has examined how structural inequalities may create unique pandemic stressors among marginalized groups.

Among college students, pandemic stressors include concerns about their own and loved ones’ health, academic performance, job loss as well as difficulty concentrating, lack of motivation, disrupted sleep, and limited social interactions [6, 7]. Prior research suggests that these stressors may be exacerbated for marginalized groups. For example, lower income students are more likely to report adverse economic and health effects of the pandemic [8]. Pre-pandemic financial strain is associated with worse economic, academic, and health effects of the pandemic among undocumented students [9]. Women-identified students were more likely than men to report disruptions of their home life and social activities [10]. Students of color reported facing and witnessing discrimination during the pandemic, which compounded the effects of other common pandemic stressors [11].

One group that may be especially exposed to pandemic stressors is undocumented immigrants. Since the onset of the pandemic, immigrant communities have been at higher risk for COVID-19 exposure and infection and have had more pandemic-related economic strain [12, 13]. This is particularly true for Latina/o/x immigrant communities which are plagued by structural racism and state-sanctioned violence that perpetuate occupational and residential segregation and higher rates of underlying health conditions, both of which condition higher COVID-related risk [14, 15]. Further, pandemic-related financial strain differs by immigration status as almost half (48%) of Latino immigrants without lawful permanent status had a hard time paying their bills during the pandemic, a much higher share than their peers with lawful permanent residency (35%) or US citizenship (26%) [16].

The ability to cope with pandemic stressors is also structurally constrained. One’s immigration status and immigrant origin can limit behavioral responses (e.g., quitting a job) and mitigating resources [17]. Federal pandemic relief efforts explicitly excluded undocumented immigrants and their families. In March 2020, Congress passed the first Coronavirus Aid, Relief, and Economic Security Act (CARES Act) which provided cash relief to eligible adults; however, undocumented immigrants and their lawfully present family members were excluded [18]. A month later, the Department of Education released guidance excluding undocumented college students from receiving emergency educational grant aid [19]. While California, where this study was conducted, provided financial support to undocumented immigrants affected by COVID-19 in May 2020, only a limited number of undocumented immigrants received funds [20]. Pre-existing state and federal policies also limited undocumented immigrants’ access to other social safety nets, such as health care and unemployment benefits [21, 22].

High rates of undocumented immigration status among Latinas/os/xs elevate their structural risk for COVID-related health disparities. Specifically, half of Mexican and Central American immigrants are undocumented [23, 24] and approximately a quarter of Latina/o/x US citizen children have an undocumented immigrant parent [25]. Individual citizenship status may mask the structural inequalities and familial legal vulnerability experienced by US citizens with undocumented family members. Specifically, both undocumented and US citizen young adults with undocumented parents may face constrained resources because they share experiences of family-level legal vulnerability such as financial insecurity [26]. Indeed, prior research has documented the convergence of educational and wellbeing outcomes among college students who are undocumented and US citizens with undocumented parents [27].

Despite elevated risk for experiencing pandemic stressors and constrained resources, prior research suggests that undocumented students and US citizens with undocumented parents are not faring particularly worse in COVID-related physical and mental health outcomes than their US-born counterparts with lawfully present parents [28]. Building on this research, we focus on Latinas/os/xs because this group may experience a unique relationship between self/parental immigration status and COVID-related mental health outcomes due to racist-nativist discourse that casts Latinas/os/xs as undocumented immigrants who are COVID-19 carriers and undeserving of aid [29, 30]. Latinas/os/xs also have higher age-specific death rates for COVID-19 compared to Whites and, in California, are 8.1 times more likely to live in households at high risk of exposure (23.6% versus 2.9%) and are overrepresented in caseloads (3,784 per 100,000 people versus 1,112) [15, 31]; this suggests that Latinas/os/xs may experience unique pandemic-related stressors that may strain their mental health. Second, we take a mixed-methods approach because similar outcomes may be concealing unique stress and coping processes among undocumented students and students with undocumented parents. Pearlin’s stress process theory acknowledges the structural nature of stressors and coping responses, such as low socioeconomic status, racial/ethnic minoritization, and undocumented immigration status, which not only increase exposure to certain stressors, but also constrain one’s ability to respond to them [32]. Other work has found that undocumented students experience stressors common among young adults (e.g., fear of the future, academic pressure) in ways that are uniquely informed by the precarity of their or their families’ immigration statuses [33]. For undocumented students and students with undocumented parents, legal vulnerabilities may prompt unique pandemic stressors as well as constrain coping resources in ways that quantitative data alone may not capture.

This paper examines whether and how self and parental immigration status contributes to Latina/o/x college students’ mental health and pandemic stressors. We draw on quantitative and qualitative survey data from 1,600 Latina/o/x University of California (UC) undergraduate students split between three groups: undocumented students, US citizens with at least one undocumented parent, and US citizens with lawfully present parents. We address three research questions: (1) Do pandemic-related negative mental health effects of Latina/o/x college students differ by self/parental immigration status?; (2) What pandemic stressors did Latina/o/x college students from immigrant families experience, and do these differ by self and parental immigration status?; and (3) How does self and parental immigration status affect their management of pandemic stressors? Prior research has established that parental undocumented status spills over into the lives of their US citizen children to compromise their overall wellbeing, including financial stability, mental health, and potential for future upward mobility [26]. Thus, we hypothesize that Latina/o/x undocumented students and US citizens with undocumented parents will report worse mental health impacts, unique stressors, and fewer resources to aid them in managing these stressors when compared to Latina/o/x US citizens with lawfully present parents.

## Methods

We draw data from a survey fielded from March to June 2020 with undergraduate students from immigrant families across California. The eligibility criteria included being over age 18, having at least one immigrant parent, and current enrollment as a UC undergraduate student. Respondents were recruited from all nine UC undergraduate campuses via emails from each campus’s undocumented student support services office, faculty teaching large general education and ethnic studies courses, and departmental and university newsletters. The survey was administered via Qualtrics with an estimated completion time of 25–35 min. Respondents provided informed consent through an information sheet and consent question displayed at the beginning of the survey. Respondents received a $10 electronic gift card as compensation for their time. All project activities were approved by the University of California, Irvine IRB.

The survey was fielded to answer research questions related to how immigration-related policies produce educational and wellbeing inequities; however, due to the evolving nature of the pandemic, respondents were asked three questions related to the COVID-19 pandemic beginning March 30. We focus on 1,600 Latina/o/x respondents who were administered these COVID-related questions. This includes 545 undocumented students, 543 US citizens with at least one undocumented parent, and 512 US citizens with lawfully present parents.

### Quantitative Analysis

To assess differences in pandemic produced negative mental health effects by self and immigration status (RQ1), we conducted bivariate analyses and multivariate linear regression models to test whether COVID-19 impacts on mental health differed by self/parental immigration status. The dependent variable was measured continuously with the following question: “To what extent has your mental health been negatively affected by the COVID-19/coronavirus pandemic.” Response options included “not at all” (0), “a little” (1), “a moderate amount” (2), “a lot” (3), and “a great deal” (4). The independent variable was self/parental immigration status which was self-reported for the student and up to two parent/guardians to identify the three comparison groups: (1) undocumented students, (2) US citizen students with at least one undocumented parent, (3) US citizen students with lawfully present parents (reference group). Control variables included gender, age, year in school, household income, and campus. Our analytic sample was 1,520 after list-wise deletion of cases with missing data. All analyses were conducted using Stata 17.

### Qualitative Analysis

To identify pandemic stressors (RQ2), we analyzed responses to a write-in question: “Please describe how the COVID-19/coronavirus pandemic has affected you and your family.” Responses ranged from a few words to multiple paragraphs; most responses were a few sentences. There was a 77.9% response rate for an analytic sample of 1,247. We analyzed these responses using flexible coding techniques [34]. First, index codes were developed to enable data reduction and retrieval (e.g., financial consequences, academic consequences, mental health consequences); these were independently applied by two research assistants and discrepancies were adjudicated by a third research assistant. The authors identified four types of stressors after multiple readings of the responses and identified analytic sub-codes within each type (see Table 1). These represent *code families*, where several sub-codes reflect distinct aspects of a general theme [35]. Index codes were used to restrict the data to relevant sections (e.g., financial consequences index code was used to restrict data for coding financial strain stressors). All analytic codes were applied independently by two coders, with a third adjudicating discrepancies. We tabulated the frequency of each analytic code and compared the frequency and substantive content of each across the three self/parental immigration status comparison groups.

**Table 1:** Description of analytic codes

Pandemic stressor codes	Description
* Financial strain*	
Job loss(es)	Mentions one or more job losses, either their own or a member of their household.
Reduced hours	Mentions reduced hours at work, either for themselves or a member of their household.
Fear of future job loss(es)	Mentions fear of future job loss(es), either for themselves or a member of their household.
Material deprivation	Mentions material deprivation such as food insecurity (e.g. rationing food, not enough food, accessing free school lunches), housing insecurity, or inability to provide for basic needs.
Limited safety net	Mentions limited access to safety nets to address income loss, such as limited savings, no federal relief (e.g. stimulus check ineligibility), no unemployment benefits, limited access to social services (e.g. food stamps).
Financial pressure	Mentions increased financial responsibilities and/or pressure to contribute to household income, such as working more hours, being the primary person employed, taking on second job, or paying additional rent/bills.
* Academic strain*	
Remote learning challenges	Mentions academic strains related to general remote learning challenges, such as difficulties learning in an online format, technology or internet issues, and lack of access to campus resources.
Limited study space	Mentions academic strains related to limited study space, such as crowded households, little to no private personal study space, and noisy environments.
Academic consequences of increased family responsibilities	Mentions academic strains related to increased family responsibilities, such as caring for family members, supporting others’ remote education, and household chores. Does not include financial responsibilities captured by financial pressure.
* COVID-19 virus concerns*	
Exposure risk	Mentions concerns about risking exposure to the virus, such as by continued work as an “essential worker” or being unable to maintain social distance in certain environments.
Infection fears	Mentions concerns about infection or virus contraction, such as cascading consequences of infection, compromised healthcare access, underlying conditions, and testing positive.
* Social dynamics*	
Strained family relationships	Mentions strained family relationships due to pandemic conditions, such as worsening family conflicts, or being forced to remain in toxic and/or unsafe household environments.
Social isolation	Mentions social isolation due to pandemic conditions, such as limited contact with family, friends, or members of the campus community (e.g. professors, staff, peers).
Immigration status effect codes	
No CARES Act stimulus checks	Mentions ineligibility for stimulus checks provided by the federal CARES Acts, including phrases like the $1200, stimulus package, stimulus check, government relief, and federal aid.
No CARES Act education relief grants	Mentions ineligibility for education relief grant provided by the federal CARES Act.
No unemployment benefits	Mentions own or family members’ ineligibility for unemployment benefits, including phrases like unemployment insurance and unemployment payments.
Limited access to social safety net programs	Mentions own or family members’ ineligibility or limited access to social safety net programs, including phrases like Cal Fresh, welfare, food stamps, and EBT.
Limited access to healthcare	Mentions own or family members’ ineligibility or limited access to healthcare, including no health insurance, unaffordability of healthcare, poor healthcare services, and immigration-related concerns with accessing healthcare services.

To examine how students’ self and family immigration status affected their management of pandemic stressors (RQ3), we analyzed responses to a qualitative write-in question: “Has your or your family members’ immigrant origin affected your experience of the COVID-19/ coronavirus pandemic? If so, please describe how.” Responses ranged from one sentence to one paragraph; most responses were a few sentences. There was a 59.6% response rate (*n*=955). Responses were index coded for types of immigration-related effects (e.g., immigration status, immigrant origin, race/ethnicity). We isolated cases that reported immigration status-related effects (*n*=439) and developed five analytic codes (see Table 1). All analytic codes were applied independently by two coders with a third adjudicating discrepancies. Finally, we tabulated the frequency of each analytic code and compared the frequency and substantive content of each across the three self/parental immigration status comparison groups.

## Results

### Negative Mental Health Effects of the Pandemic

Table 2 provides descriptive statistics of our full sample. About three-quarters of the undocumented respondents were Deferred Action for Childhood Arrivals (DACA) recipients, with most of the remainder having no current legal status. The vast majority of undocumented respondents had at least one undocumented parent/guardian, and three-quarters of US citizens from mixed-status families reported that all their parents/guardians were undocumented. The majority reported Mexico as their country of origin. About three-quarters of respondents identified as women and almost all were adults in their early 20s. 22.3% were in their first year, 18.1% in their second, 28.6% in their third, and 30.9% in their fourth or more year. About a quarter of undocumented students and US citizen students with undocumented parents reported their household income as less than $20,000 a year; about half that number of US citizen students with lawfully present parents reported the same. The vast majority of participants reported negative mental health effects related to the pandemic. 92.9% of all respondents reported that their mental health had been negatively affected by the pandemic, with 25.4% reporting this “a lot” and 26.9% “a great deal.”

**Table 2:** Demographic characteristics of participants (*n*=1600)

	Undocumented students (*n*=545)		US citizens with at least one undocumented parent (*n*=543)		US citizens whose parents are lawfully present (*n*=512)		Total (*n*=1,600)	
	*n*	% or mean (SD)	*n*	% or mean (SD)	*n*	% or mean (SD)	*n*	% or mean (SD)
**Student immigration status**								
US citizen	-	0.0	543	100.0	512	100.0	1055	65.9
Undocumented—DACA	399	73.2	-	0.0	-	0.0	399	24.9
Undocumented—other liminal legal status	7	1.3	-	0.0	-	0.0	7	0.4
Undocumented—no legal status	139	25.5	-	0.0	-	0.0	139	8.7
**Student area of origin**								
Mexico	457	83.9	-	0.0	-	0.0	457	28.6
Central America	63	11.6	-	0.0	-	0.0	63	3.9
South America	22	4.0	-	0.0	-	0.0	22	1.4
Other	3	0.6	-	0.0	-	0.0	3	0.2
USA	-	0.0	543	100.0	512	100.0	1055	65.9
**Parental immigration status**								
Undocumented	476	87.3	391	72.0	-	-	867	54.2
LPR	12	2.2	-	0.0	114	22.3	126	7.9
US citizens	3	0.6	1	0.2	237	46.3	241	15.1
Undocumented and LPR	10	1.8	72	13.3	-	-	82	5.1
Undocumented and US citizen	5	0.9	51	9.4	-	-	56	3.5
LPR and US citizen	1	0.2	-	0.0	151	29.5	152	9.5
Other combinations	12	2.2	17	3.1	5	1.0	34	2.1
Missing	26	4.8	11	2.0	5	1.0	42	2.6
**Has one US born parent**								
No	544	99.8	539	99.3	468	91.4	1551	96.9
Yes	1	0.2	4	0.7	44	8.6	49	3.1
**Parent/guardian 1 area of origin**								
Mexico	455	83.5	475	87.5	387	75.6	1317	82.3
Central America	64	11.7	55	10.1	69	13.5	188	11.8
South America	23	4.2	2	0.4	14	2.7	39	2.4
Other	2	0.4	1	0.2	10	2.0	13	0.8
USA	1	0.2	10	1.8	32	6.3	43	2.7
**Parent/guardian 2 area of origin**								
Mexico	346	63.5	423	77.9	312	60.9	1081	67.6
Central America	44	8.1	44	8.1	58	11.3	146	9.1
South America	21	3.9	2	0.4	12	2.3	35	2.2
Caribbean	-	0.0	-	0.0	1	0.2	1	0.1
Other	-	0.0	1	0.2	10	2.0	11	0.7
USA	1	0.2	5	0.9	45	8.8	51	3.2
Does not report second parent	133	24.4	68	12.5	74	14.5	275	17.2
**Gender**								
Female	399	73.2	434	79.9	381	74.4	1214	75.9
Male	129	23.7	102	18.8	117	22.9	348	21.8
Non-binary, queer, transgender	16	2.9	6	1.1	12	2.3	34	2.1
Missing	1	0.2	1	0.2	2	0.4	4	0.3
**Age**								
18–20	257	47.2	328	60.4	269	52.5	854	53.4
21–23	229	42.0	196	36.1	213	41.6	638	39.9
24 and older	59	10.8	19	3.5	30	5.9	108	6.8
**Year in school**								
First year undergraduate	101	18.5	145	26.7	110	21.5	356	22.3
Second year undergraduate	86	15.8	108	19.9	96	18.8	290	18.1
Third year undergraduate	174	31.9	141	26.0	143	27.9	458	28.6
Fourth year undergraduate	148	27.2	122	22.5	139	27.2	409	25.6
Fifth year or more undergraduate	36	6.6	26	4.8	23	4.5	85	5.3
Missing	-	0.0	1	0.2	1	0.2	2	0.1
**Household income**								
Less than $20,000	132	24.2	134	24.7	66	12.9	332	20.8
$20,001 to $40,000	228	41.8	204	37.6	135	26.4	567	35.4
$40,001 to $75,000	129	23.7	146	26.9	182	35.6	457	28.6
Greater than $75,001	31	5.7	43	7.9	101	19.7	175	10.9
Missing	25	4.6	16	3.0	28	5.5	69	4.3
**Extent COVID-19 negatively impacted mental health**		2.59 (1.24)		2.41 (1.21)		2.47 (1.26)		2.49 (1.24)
Not at all	35	6.4	32	5.9	38	7.4	105	6.6
A little	81	14.9	111	20.4	85	16.6	277	17.3
A moderate amount	124	22.8	126	23.2	122	23.8	372	23.3
A lot	136	25.0	147	27.1	124	24.2	407	25.4
A great deal	167	30.6	126	23.2	138	27.0	431	26.9
Missing	2	0.4	1	0.2	5	1.0	8	0.5

Table 3 shows the multivariate regression results comparing COVID-19 impacts on mental health by self/parental immigration status. The mean value for COVID-related mental health problems for the reference category was 2.46 (i.e., between “moderate” and “a lot”). The mean was 2.59 for undocumented students and 2.41 for US citizens with undocumented parents. These differences were not statistically significant, however.

**Table 3:** Multivariate linear regression of COVID-19 impacts on mental health (*n*=1,520)

	*β*	95% CI		*p*
Self/parental immigration status				
US citizens with lawfully present parents	Referent	-	-	-
Undocumented students	0.08	−0.07	0.24	
US citizens with undocumented parents	−0.06	−0.21	0.09	
Gender				
Men	Referent	-	-	-
Women	−0.64	−0.79	−0.50	**
Non-binary, queer, transgender	0.57	0.15	1.00	*
Age				
18–20	Referent	-	-	-
21–23	0.09	−0.12	0.29	
24 and older	0.03	−0.27	0.34	
Year in school				
First year undergraduate	Referent	-	-	-
Second year undergraduate	0.11	−0.07	0.30	
Third year undergraduate	0.28	0.08	0.48	*
Fourth year undergraduate	0.26	−0.01	0.52	
Fifth year or more undergraduate	0.38	0.02	0.73	*
Household income				
Less than $20,000	Referent	-	-	-
$20,001 to $40,000	−0.15	−0.31	0.01	
$40,001 to $75,000	−0.14	−0.31	0.03	
Greater than $75,001	−0.32	−0.55	−0.10	*
Campus				
UC Berkeley	Referent	-	-	-
UC Davis	−0.19	−0.49	0.11	
UC Irvine	−0.49	−0.77	−0.21	*
UC Los Angeles	−0.28	−0.57	0.02	
UC Merced	−0.64	−0.93	−0.35	**
UC Riverside	−0.37	−0.64	−0.09	*
UC Santa Barbara	−0.13	−0.42	0.17	
UC Santa Cruz	−0.13	−0.42	0.15	
UC San Diego	−0.30	−0.64	0.05	

Notes: *p < 0.05; **p < 0.001

### Pandemic Stressors

Our qualitative analysis revealed four types of pandemic stressors: financial insecurity, COVID-19 virus concerns, academic strains, and social dynamics. Table 4 reports the frequency of these code families and the analytic codes. Given the broad nature of the survey question and the relatively short response length, these do not represent prevalence rates of the stressors; respondents likely had experiences that they did not report. Thus, our analysis focuses on comparing the relative frequency of a code and substantive content differences across the three groups.

**Table 4:** Frequency of pandemic stressors by self/parental immigrations status

Stressors	Undocumented students (*n*=438)	US citizens with undocumented parents (*n*=433)	US citizens with lawfully present parents (*n*=376)
Financial insecurity	70.1%	70.0%	49.5%
Job loss(es)	37.%	42.0%	29.0%
Reduced hours	26.3%	26.6%	16.0%
Fear of future job loss(es)	5.0%	3.0%	5.1%
Material deprivation	12.8%	9.5%	6.6%
Limited safety net	11.9%	9.2%	3.7%
Financial pressure	7.1%	6.2%	3.5%
COVID-19 virus concerns	17.1%	14.8%	19.4%
Exposure risk	10.3%	10.2%	12.5%
Infection fears	8.9%	8.1%	10.6%
Academic strains	20.1%	19.2%	16.0%
General remote learning challenges	14.6%	14.1%	12.8%
Limited study space	4.6%	3.7%	3.2%
Increased family responsibilities	4.6%	4.4%	2.7%
Social dynamics	4.8%	5.1%	6.6%
Strained family relationships	3.2%	2.5%	4.0%
Social isolation	1.6%	2.5%	2.7%

#### Financial Insecurity

The most common stressor was income loss associated with the economic impacts of the pandemic, including job loss and reduced work hours. Students from all three groups focused on the depth of COVID-19’s impact on their own and their family’s employment with responses like the following:

“I lost both my jobs and had to move out of my apartment since I could no longer afford to live there.” (*Undocumented student*)

“One family member has been laid off. Another one works less often. The head of my household may be at risk and is noticing less work at the office.” (*US citizen with undocumented parents)*

“My parent’s job has cut their hours significantly. My dad lost one of his jobs and is only working a part-time job at the moment.” (*US citizen with lawfully present parents*)

Notably, undocumented students and citizens with undocumented parents reported job and income loss more frequently than US citizens with lawfully present parents. Further, many tied this to their employment in the service sector which was significantly curtailed due to California’s stay-at-home orders. A small portion of students in all three groups mentioned fear of future job loss.

A second dimension of financial insecurity stressors was related to ensuing material deprivation, including food insecurity and an inability to meet basic needs. For example, one US citizen with lawfully present parents wrote, “COVID-19 has taken our jobs away making it harder to pay bills and obtain basic needs.” These comments arose among all three groups but were more common among the undocumented students, followed by US citizens with undocumented parents. Further, undocumented students highlighted their deprivation, rather than more generalized financial difficulties. For example, one wrote, “Some of my family no longer has a job and are struggling to purchase food and not be evicted from their home” and another shared, “My family is rationing food, we are unable to pay our rent or bills.”

A third dimension of financial insecurity stressors was having a limited safety net to manage economic strains. For example, one US citizen with lawfully present parents wrote, “My mother can be laid off [from] her work at any time, and this would leave us with nothing as we cannot afford to save money each paycheck.” Such comments arose among all three groups. In addition, undocumented students and US citizens with undocumented parents highlighted their ineligibility for social services and federal relief aid that would help cushion the blow of income loss. “My parents and I were laid off from our jobs. We live by a daily check and we do not qualify for the stimulus check nor for any government assistance.”

A final dimension of financial insecurity stressors manifested as increased pressure and responsibility to help cover household expenses. This stressor was more common among undocumented students and US citizens with undocumented parents who emphasized they had become the sole financial providers for their family or took on additional jobs:

“If I stop working that means less income to pay for bills, food, and housing. That if I stop working and my dad does not get paid on time we don’t have the money necessary to be able to buy essential items.” (*Undocumented student*)

“I had to take another job on top of the one I have in order to provide help for my family, as their jobs are day to day and my sister was let go.” (*US citizen with undocumented parents*)

Fewer US citizens with lawfully present parents noted these types of increased responsibilities and those who did reported less pressing demands. For example, one wrote, “I know my parents have savings but I know they are getting depleted so I feel pressured to start working and contributing money in the household.”

#### COVID-19 Virus Concerns

One dimension of COVID-19 virus stressors was related to exposure risk, primarily related to students’ and parents’ classification as “essential workers.” Students from all three groups shared concerns about exposure to the virus, mostly as part of their employment:

“For me, as an essential worker, I have had to work a lot more hours. I also have to deal with the stress of being around over 200 people a day.” (*Undocumented student)*

“My family still has to go to work and we rely on public transportation, and with limited resources, it is difficult to social distance and stay safe.” (*US citizen with undocumented parent*)

“My father gets up everyday worried about the virus since he is a truck driver and he doesn’t want the guilt of infecting us with it.” (*US citizen with lawfully present parents*)

Although students with lawfully present parents were slightly more likely to identify exposure related stressors, they also identified a wider range of exposure sources such as working in healthcare settings or “going outside.” Undocumented students and US citizens with undocumented parents focused more on feeling forced to take such risks in order to financially sustain their family’s wellbeing with one undocumented student commenting, “I feel pain because my community fears more for losing their jobs than their health safety.”

A second dimension of COVID-19 virus stressors was fear of the consequences of the student or their family members being infected. Students in all three groups shared such concerns but undocumented students and US citizens with undocumented parents tended to express concerns for cascading infection risk in overcrowded living situations and multi-family households as well as limited access to healthcare due to immigration status:

“We’re stuck in a small one-bedroom apartment for social distancing, so I fear if one of us gets sick, we’ll all get sick and not be able to afford health care due to our status and income.” *(Undocumented student)*

“My family and I have to be extra careful to not get infected because we babysit my 2-year-old nephew while his parents go to work since they are essential workers and also being careful because my mom has diabetes and is at risk if she gets infected.” *(Undocumented student)*

“[I] worry that I could be a carrier and get my mom sick. Since my mom is undocumented and does not qualify for health insurance …, she would not be able to receive healthcare. I worry that this could threaten her life. I also worry because she has anxiety and this pandemic has been triggering her. If her anxiety gets worse, she would not be able to seek professional help because she is uninsured and we do not have the money to pay for the services.” *(US citizen with undocumented parent)*

By contrast, US citizens with lawfully present parents highlighted pre-existing health conditions that placed them and their family at higher risk for infection and complications. For example, “The pandemic has created some nervousness and worry in my family because a lot of us are immunocompromised and my dad is currently an essential worker, so we take extra precautions.”

#### Academic Strains

Academic stressors were slightly more frequent among undocumented students and US citizens with undocumented parents. The primary academic stressor was general strain related to remote learning. Students recounted difficulties with the new instruction mode:

“Online schooling just is not for me. It is just so hard for me to concentrate and learn through a computer.” *(Undocumented student)*

“The move to online is not manageable. We have poor internet and can’t get better internet because of our location and because it’s too expensive.” *(US citizen with undocumented parents)*

I feel suffocated by my school work and professors don’t seem to have empathy for students. (*US citizen with lawfully present parents*)

Two additional academic stressors aggravated the more common remote learning stressors: limited space and increased family responsibilities. Students who lived in overcrowded housing conditions reported a lack of privacy, too much noise, and inadequate study space as additional stressors that made remote learning particularly difficult:

“The struggle of being back home - I don’t have a bed, desk, closet, or space for myself living in a very small 2 bedroom apartment with a total of 6 people in my household it is very hard to get my school work done.” *(Undocumented student)*

“It is hard to be in a house where loudness is a constant and having to study through it is quite difficult.” *(US citizen with undocumented parents)*

Additionally, increased family responsibilities compromised students’ academics. For example, an undocumented student wrote, “My life these past months in quarantine has solely been taking care of my siblings; I feed them, clean the apartment, deal with their difficult tempers, and make sure they are doing schoolwork.” Most commonly, family responsibilities included providing childcare and remote learning support for siblings as well as household chores. These academic distractions were slightly more pronounced among undocumented students and US citizens with undocumented parents.

#### Social Dynamics

One dimension of social dynamic stressors was related to family relationships. Students from all three groups spoke of returning to their family homes and having to confront existing family conflicts and “toxic” environments with negative implications for their mental health:

“My family has a long history of domestic violence, so I honestly do not feel safe being back home. I keep waiting for things to explode, this is not helping my mental health.” *(Undocumented student)*

“My parents’ constant arguing affects my mental health and I live in stress and nervousness because I never know what will happen at home.” *(US citizen with undocumented parents)*

“Mental health has been a struggle, I am currently in the closet from my father who is very homophobic. I sometimes struggle to be myself around him. I have not been in this house this long with him since high school.” *(US citizen with lawfully present parents)*

Differences emerge by self/parental status as undocumented students and citizens with undocumented parents were more likely to report “crammed,” “cooped up,” and “confined” living quarters, which compromised family relationships and strained their mental health:

“Because we all have to shelter in place, there is no privacy or alone time, something I am incredibly accustomed to, increasing general anxiety, nervousness, and irritability” *(Undocumented student)*

“Everyone in the household is very irritable due to the close contact… terrible sleep and eating schedule” *(US citizen with undocumented parents)*

These two student groups mostly focused on the lack of physical space in their households and family conflicts that arose from living closely together.

A second dimension of social dynamic stressors was social isolation due to stay-at-home orders. All three student groups noted social isolation felt from not being able to see or interact with others outside of their household. However, undocumented students and US citizen students with undocumented parents were more likely to note social isolation felt from not being able to interact in-person with the campus community:

“I do not feel as connected to some of my professors. I have withdrawn socially since it was automatically there when I lived on campus and now it requires extra effort so I am working on it, but it took a toll on my mental health. My stress and anxiety levels have risen since I do not remember/feel I have access to TAs or tutoring services despite the fact that some are available online still.” *(Undocumented student)*

“My housemates who are good friends to me are in [another part of California] … I feel I lost a big support system and my daily routine. Friends are dispersed and it can feel very alienating.” *(US citizen with undocumented parents)*

Citizens with lawfully present parents were more likely to speak about social isolation as a family unit: “Staying at home has clearly made both of our mental health decline. I can tell my mom sulks a lot not being able to be out and the longer we’re in isolation the more I retreat and return to my bad eating habits and tendency to watch Netflix and distance myself from friends.”

### Managing Pandemic Stressors

Our analysis revealed five ways that immigration status affected students’ ability to manage pandemic stressors: no stimulus checks, no unemployment benefits, limited access to healthcare, limited access to social safety net programs, and no education relief grants. Table 5 reports the frequency of these analytic codes. Nearly half (51.2%, *n*=173) of undocumented students reported one of these forms of immigration status-related exclusion from resources that would aid them. US citizens followed closely behind with 41.7% (*n*=150) reporting this, often linking their fate with those of their undocumented family members. In a few cases (5.1%, *n*=13), US citizens with lawfully present parents pointed to their undocumented extended family members’ exclusion.

**Table 5:** Frequency of immigration status effect codes by self/parental immigration status

	Undocumented students (*n*=338)	US citizens with undocumented parents (*n*=360)	US citizens with lawfully present parents (*n*=257)
No CARES Act stimulus checks	34.9%	30.8%	3.1%
No unemployment benefits	12.7%	6.1%	0.8%
Limited access to healthcare	8.9%	6.9%	1.6%
Limited access to social safety net programs	3.3%	1.4%	0.0%
No CARES Act education relief grants	1.5%	0.0%	0.0%
Total reported one or more immigration status effect code	51.2%	41.7%	5.1%

Overwhelmingly, undocumented students and citizens with undocumented parents cited their exclusion from federal coronavirus relief efforts when asked if their own or family members’ immigrant origin affected their pandemic experiences. For example, an undocumented student wrote, “My parents and I being undocumented has affected our lives during this pandemic because we are not getting any help from the government. My little sisters are both US citizens but they’re essentially being forgotten about for having immigrant parents.” Both groups highlighted how undocumented immigrants and their US citizen family members were excluded from this economic relief. A few undocumented students also noted that they were not eligible for emergency education grants due to their immigration status.

Undocumented students, followed by US citizens with undocumented parents, also cited their exclusion from pre-existing social safety nets because of their undocumented status, which endangered their ability to successfully manage financial stressors.

“Being [undocumented] immigrants means none of us are eligible for unemployment. This is something difficult to see as others in our same jobs are eligible and seeing them get unemployment checks is a reminder of our marginality” (*Undocumented student*)

“My parents also cannot apply for unemployment or food stamps because all are undocumented, and do not want to be a public charge.” (*US citizen with undocumented parents*)

Ineligibility for unemployment benefits was mentioned by about one in five undocumented students and one in ten citizens with undocumented parents. Other social services, specifically government-funded food access programs, were mentioned less frequently.

Lastly, about one in seven undocumented students and one in ten citizens with undocumented parents noted that ineligibility for health insurance exacerbated virus stressors. One citizen with undocumented parents wrote, “I simply worry that if my parents get sick we won’t be able to afford the correct medical treatment necessary since they do not have insurance due to their legal status.” Additionally, these students spoke to the fear of experiencing unfair treatment, discrimination, and deportation risk if they interacted with the healthcare system:

“We feel that around this time, ICE and such organizations will be more present and hospitals and healthcare facilities” (*Undocumented student*)

“I fear that if one of my parents … contract the coronavirus, they will be denied medical service or given poor service because of their legal status… They also do not have health insurance, so on top of not being treated fairly, they will have an immense bill to pay if they are treated in a hospital” (*US citizen with undocumented parents*).

## Discussion

We examined whether self and parental immigration status contributed to differences in Latina/o/x college students’ mental health and pandemic stressors during the initial months of the COVID-19 pandemic. Quantitative analyses revealed that the pandemic produced widespread negative mental health effects with no differences by self/parental immigration status for this group. Qualitative analyses identified four types of pandemic stressors: financial insecurity, COVID-19 virus concerns, academic strains, and social dynamics. Comparisons across self/parental immigration status revealed that many stressors are shared; however, undocumented students and US citizens with undocumented parents reported some stressors more frequently and highlighted unique aspects of others. Finally, self and parental immigration status was salient in students’ perceptions of their ability to manage these pandemic stressors because undocumented status prevented them from accessing necessary resources.

We found high levels of negative mental health effects due to the pandemic. Nearly all students reported that their health was negatively affected by the pandemic with slightly more than half reporting “a lot” or “a great deal.” Regression analyses revealed no statistically significant group differences in the severity of effects. Thus, despite unique stressors, it does not appear that Latina/o/x undocumented students and US citizens with undocumented parents fared worse in their mental health effects. These findings are consistent with a prior study which found no group differences among a racially diverse sample [28]. This suggests that while Latinas/os/xs may have been experiencing unique racist-nativist discourses and disproportionate stressors during the pandemic, these did not alter the relationship between self/family immigration-status and mental health effects of the pandemic. Another reason for a lack of group differences may be that undocumented students and US citizens with undocumented parents experienced stress inoculation as they already contended with related stressors prior to the pandemic due to their legal vulnerability [28, 36]. Indeed, prior research suggests that undocumented students normalize their mental health strain [37]. Finally, given the sudden social shock inflicted by the pandemic and the drastic changes it caused to everyday life, it stands to reason that all three student groups would report elevated negative mental health effects. Our qualitative data suggests that some stressors, such as those related to remote learning and social dynamics, were mentioned with similar frequency across the three groups of students. These shared experiences with remote learning and social isolation indicate shared sources of stress as students were all forced to remain in their households due to safer-at-home orders.

Despite this, self and parental immigration status does appear to differentiate the pandemic stressors experienced by students. Financial insecurity stressors were by far the most common across all three groups, reflecting the immediate and severe economic turmoil caused by the pandemic. Notably, this stressor was substantially more frequent among undocumented students and US citizens with undocumented parents, particularly as it relates to job loss and reduced work hours. Undocumented students, followed by US citizens with undocumented parents, were also more likely to report material deprivation and having a limited safety net to weather income loss. The similarities between these two groups stem from the reality that they share legal vulnerabilities at the family level. Specifically, both groups have undocumented parents and family members who have precarious employment due to a lack of work authorization [38]. Further, undocumented workers are overrepresented in industries that were shut down and/or curtailed by stay-at-home orders meant to slow the spread of the disease, compromising their potential to earn income [39], [40]. Indeed, undocumented men saw the steepest increase in job loss compared to US-born and documented immigrant men, climbing from 4.5% pre-pandemic to 31.9% in spring 2020 [41]. Further, undocumented immigrants earn less than their documented peers pre-pandemic, increasing the likelihood that they did not have the savings needed to weather economic losses [38]. Although this stressor was less frequent among US citizens with lawfully present parents, almost half reported at least one financial insecurity stressor. This reflects the reality that citizens with lawfully present parents can also be members of low-income families given the persistence of a substantial wage gap for Latina/o/x immigrants more broadly [42]. Thus, while stressors may be shared across groups in this study, the source of the stressor may be traced back to different types of structural vulnerabilities.

Self and parental immigration status also contributed to legal vulnerabilities that prompted unique stressors among undocumented students and US citizens with undocumented parents. For example, virus concerns highlighted unique employment-related exposure risk. Indeed, estimates reveal that nearly three in four undocumented workers held “essential” jobs at the beginning of the pandemic, suggesting that they were at increased risk of exposure to the novel coronavirus [43]. Further, infection fears established cascading infection concerns due to cramped living situations and limited healthcare access due to undocumented status. Although remote learning struggles and disruptive family dynamics were reported by all three student groups, undocumented students and citizens with undocumented parents faced additional academic and social stressors related to crowded living situations and being cut off from their university campuses. Prior research has established that undocumented immigrants and their families are more likely to live in overcrowded living situations [44]. This reality translated into limited access to quiet study space and constrained ability to escape toxic family dynamics.

Prior research has established that undocumented immigration status is one of many fundamental causes of health disparities because it constrains access to resources that can protect one’s health [17]. Building on this body of literature, we show how the marginalized social status of undocumented immigrants contributed to the proliferation of pandemic stressors. Further, the exclusion of undocumented immigrants and mixed-status families from relief efforts and social services directly constrained the resources available to manage stressors. Most prominently, students highlighted how federal pandemic relief excluded undocumented immigrants and their citizen family members from receiving aid, explicitly denying them access to financial resources needed to help weather the economic crisis. Further, undocumented immigrants’ exclusion from social safety nets (e.g., unemployment benefits, government food assistance programs) and limited healthcare access compromised students’ and family’s ability to access resources to mitigate financial and infection stressors.

Overall, the findings highlight the importance of examining underlying processes as disparities may not be immediately evident in health outcomes. As illustrated by the quantitative analysis, mental health outcomes were extremely high in the early months of the pandemic for all groups. However, the qualitative data uncovers which stressors are shared among all students and which are unique to undocumented students and US citizens with undocumented parents. Notably, many of the shared stressors—remote education and social isolation—have been addressed with a return to in-person experiences due to the development of vaccines and better public health knowledge. However, the stressors most common among students who are undocumented or have undocumented parents—financial strains, virus exposure, and access to healthcare—are likely to persist for a longer period as communities continue to reel from the pandemic’s long-term economic and health effects. Given these differences, it may be that immigration-related mental health disparities may emerge in later phases of the pandemic as initial disruptions recede but longer-term consequences persist.

Our study had some limitations. Foremost, our qualitative data is composed of short-answer responses to two write-in survey questions. We do not ask specifically about individuals’ mental health stressors and so are also unable to infer the prevalence of each stressor because we did not systematically ask about each type of stressor. The brief responses suggest that respondents focused on a few of the most immediate stressors, possibly contributing to the low frequency of some stressors. The brief responses also limit our ability to ascertain details about the processes through which these stressors affect students’ mental health. Secondly, about three-quarters of the undocumented students reported being DACA recipients and may not adequately document the depth of legal vulnerabilities experienced by undocumented students with no legal status. Finally, our study took place in California which is a relatively inclusive policy context for undocumented immigrants; stressors related to legal vulnerability may be distinct in other policy contexts.

## Conclusions

Our study examines the COVID-19 pandemic’s early toll on the mental health of a vulnerable and understudied population: Latina/o/x children of immigrants with varying self and parental immigration status. It draws on a novel large-scale survey that is the first we are aware of to collect detailed personal and parental information and COVID-related outcomes in this population. Although the mental health outcomes do not indicate elevated risks for Latina/o/x undocumented students and US citizens with undocumented parents, we find that their stressors and coping resources are unique. This suggests that the high-stress nature of the pandemic may elevate distress across the board but the structural exclusion of certain groups contributes to unique stress processes.

Our findings indicate the importance of recognizing structural inequalities when identifying sources of stress and coping responses, especially among young people who are undocumented or have undocumented family members. Their legal vulnerabilities increased exposure to pandemic stressors, created unique types of stressors, and constrained access to resources to help alleviate stressors. While interventions aimed at managing stress may help, clinicians must be aware of and openly acknowledge the structural constraints that may limit their effectiveness. Indeed, prior research has established that mental health service seeking among undocumented students is compromised by perceived inefficacy of services to address such larger structural problems [37]. Thus, structural interventions should also be developed along with individual-level interventions aimed at improving mental health access and services. This includes policy interventions that will address underlying legal vulnerabilities and aid immigrants in protecting their health, both during and beyond the pandemic: access to social safety nets, affordable healthcare, and ultimately a pathway to legalization. Educational institutions must also support students’ mental health by increasing their access to resources and establishing policies that take into account students’ divergent access to resources, including space, technology, and time.
